# Roles of eIF3m in the tumorigenesis of triple negative breast cancer

**DOI:** 10.1186/s12935-020-01220-z

**Published:** 2020-04-29

**Authors:** Wei Han, Cong Zhang, Chun-tao Shi, Xiao-jiao Gao, Ming-hui Zhou, Qi-xiang Shao, Xiao-jun Shen, Cheng-jiang Wu, Fang Cao, Yong-wei Hu, Jian-liang Yuan, Hou-zhong Ding, Qing-hua Wang, Hao-nan Wang

**Affiliations:** 1 Department of General Surgery, Kunshan First People’s Hospital Affiliated to Jiangsu University, Kunshan Jiangsu, 215300 People’s Republic of China; 2 Department of Pharmacy, Kunshan Hospital of Traditional Chinese Medicine, Kunshan Jiangsu, 215300 People’s Republic of China; 3 Department of General Surgery, Wuxi Xishan People’s Hospital, Kunshan Wuxi Jiangsu, 214000 People’s Republic of China; 4 Department of Pathology, Kunshan First People’s Hospital Affiliated to Jiangsu University, Kunshan Jiangsu, 215300 People’s Republic of China; 5 Centralab, Kunshan First People’s Hospital Affiliated to Jiangsu University, Jiangsu, 215300 Kunshan People’s Republic of China; 6 Department of Immunology, Key Laboratory of Medical Science and Laboratory Medicine, School of Medicine, Jiangsu University, Zhenjiang Jiangsu, 212013 People’s Republic of China; 7 Department of Clinical Laboratory, The Second Affiliated Hospital of Soochow University, Suzhou Jiangsu, 215000 People’s Republic of China; 8 Oncology Department, Wuxi Fifth People’s Hospital, Wuxi Jiangsu, 214000 People’s Republic of China

**Keywords:** eIF3m, Triple negative breast cancer, Tumorigenesis, Bioinformation

## Abstract

**Background:**

Without targets, triple negative breast cancer (TNBC) has the worst prognosis in all subtypes of breast cancer (BC). Recently, eukaryotic translation initiation factor 3 m (eIF3m) has been declared to be involved in the malignant progression of various neoplasms. The aim of this study is to explore biological functions of eIF3m in TNBC.

**Methods:**

Multiple databases, including Oncomine, KM-plotter and so on, were performed to analyze prognosis and function of eIF3m in TNBC. After transfection of eIF3m-shRNA lentivirus, CCK-8, colony formation assay, cell cycle analysis, wound healing assay, transwell assays, mitochondrial membrane potential assay and cell apoptosis analysis were performed to explore the roles of eIF3m in TNBC cell bio-behaviors. In addition, western blotting was conducted to analyze the potential molecular mechanisms of eIF3m.

**Results:**

In multiple databases, up-regulated eIF3m had lower overall survival, relapse-free survival and post progression survival in BC. EIF3m expression in TNBC was obviously higher than in non-TNBC or normal breast tissues. Its expression in TNBC was positively related to differentiation, lymph node invasion and distant metastasis. After knockdown of eIF3m, cell proliferation, migration, invasion and levels of mitochondrial membrane potential of MDA-MB-231 and MDA-MB-436 were all significantly suppressed, while apoptosis rates of them were obviously increased. In addition, eIF3m could regulate cell-cycle, epithelial–mesenchymal transition and apoptosis-related proteins. Combined with public databases and RT-qPCR, 14 genes were identified to be modulated by eIF3m in the development of TNBC.

**Conclusions:**

eIF3m is an unfavorable indicator of TNBC, and plays a vital role in the process of TNBC tumorigenesis.

## Background

Breast cancer (BC), one of the most common diseases in women, has severely threatened human health and social development [1]. Pathologically, invasive breast carcinoma is characterized by at least four molecular subtypes: Luminal A, Luminal B, HER2-positive and triple negative breast cancer (TNBC) [2]. TNBC, accounting for 15–25% of all breast cancer cases, is characterized by the lack of expression of estrogen receptor (ER), progesterone receptor (PR) and c-erbB-2 (HER2) [2]. Despite well-established benefits of chemotherapy after surgery on tumor growth, TNBC still has the worst prognosis in all subtypes of BC in the result of no targeted therapy [2, 3]. Due to obscure pathogenesis of TNBC, rapid progress, recurrence and drug resistance also become obstacles in the process of anti-cancer treatment [4]. Therefore, it is urgent to understand genomics and explore mechanisms of carcinogenesis in order to improve the diagnosis and treatment of TNBC.

Protein synthesis has been considered as a critical step from gene expression to cell biological behavior [5]. Abnormal translations of oncogenes, tumor suppressors and eukaryotic translation initiation factors (EIFs) are essential for tumorigenesis [5]. Among eIFs, eukaryotic translation initiation factor 3 (eIF3) is the most complex eukaryotic translation initiation factor [6]. This complex composed of 13 subunits (eIF3a to m) is necessary for the initiation of protein synthesis [7]. Among these subunits, 5 core subunits (eIF3a, b, c, g and i) are conserved in all eukaryotes, while the rest only exist in mammal [8]. Recent studies have reported that several subunits are essential for the development of various tumors, including BC [9–11]. Notably, as the last defined subunit, eIF3m plays an indispensable role on maintaining the integrity of the eIF3 complex by stabilizing the core subunits [12]. Therefore, the ablation of eIF3m severely suppresses the translation and cancer cell proliferation [13]. In addition, eIF3m is a critical factor for cell cycle progression via regulating cell division cycle 25A (CDC25A) [13]. However, its function in BC or TNBC is not yet characterized.

In this research, eIF3 subunits were detected in breast tumors and corresponding adjacent normal breast tissues as well as public databases. After selection of suitable breast cancer cell lines with higher eIF3m expression, stable transfected cells with eIF3m knockdown were constructed for investigating the significance of eIF3m in vitro and exploring the potential pathogenesis of TNBC tumorigenesis. This article revealed possible roles of eIF3m in TNBC and provided a novel and reliable bio-marker for clinic.

## Methods

### Data mining of datasets

First, five cohorts selected from Oncomine (https://www.oncomine.org/) were used to analyze differences of eIF3m expression between breast tumors and normal breast tissues: “Curtis breast 2012” (1989 tumors, 3 benign breast neoplasms and 144 normal breast tissues), “Sorlie breast 2003” (92 tumors and 4 normal breast tissues), “Ma breast 2009” (9 tumors and 14 normal breast tissues), “Richardson breast 2006” (40 tumors and 7 normal breast tissues) and “TCGA breast 2012” (639 tumors and 111 normal breast tissues). To assess the prognostic role of eIF3m in BC, KM-plotter (http://kmplot.com/analysis/index.php?p=service&cancer=breast) and BCIP database (http://www.omicsnet.org/bcancer/database) were performed. The survival parameters included overall survival (OS), relapse-free survival (RFS) and post progression survival (PPS). BCIP database also showed co-expressed genes of eIF3m in the development of breast normal tissues and TNBC. In addition, a GEO database (GEO accession: GSE45827) consisting of four BC subtypes (29 cases of Luminal A, 30 cases of Luminal B, 30 cases of HER2-positive and 41 cases of TNBC) and breast normal-like tissues (11 cases) was conducted to investigate the expression of eIF3 subunits. Another two bioinformation databsaes, STRING (https://string-db.org/) and PDBsum entry (https://www.ebi.ac.uk/thornton-srv/databases/cgi-bin/pdbsum/GetPage.pl?pdbcode=index.html), were used for identifying the structure of eIF3 and the location of eIF3m. Furthermore, KEGG pathway (https://www.kegg.jp/) was used for exploring potential carcinogenic mechanisms of eIF3m.

### Patients and tissue specimens

Specimens from a total of 184 female invasive breast cancer patients who underwent modified radical mastectomy, breast reservation radical correction or mastectomy (only for distant metastasis) in Kunshan First People’s Hospital (63 cases), Kunshan Hospital of Traditional Chinese Medicine (37 cases), Wuxi Xishan People’s Hospital (49 cases) and Wuxi Fifth People’s Hospital (35 cases) from January, 2016 to January, 2019 were collected, with a mean age of 53.47 ± 13.02 years. According to the pathological diagnosis, specimens were divided into four subtypes: 42 Luminal A, 62 Luminal B, 43 HER2-positive and 37 TNBC. Every case consisted of breast tumor and corresponding adjacent normal tissue. No one received any radiotherapy or chemotherapy before surgery. All cases had paraffin-embedded consecutive sections that were used for Immunohistochemistry (IHC). Among them, 158 samples including 32 TNBC and 126 Non-TNBC with corresponding adjacent normal tissues were available for reverse transcription-quantitative PCR (RT-qPCR), while we failed to extract total RNA from the rest. This research had received the approval of Kunshan First People’s Hospital Ethics Committee, Kunshan Hospital of Traditional Chinese Medicine Ethics Committee, Wuxi Xishan People’s Hospital Ethics Committee and Wuxi Fifth People’s Hospital Ethics Committee. Every patient signed the informed consent form.

### Cell lines and cell culture

Five human breast cancer cell lines (SK-BR3, MCF-7, BT-474, MDA-MB-231 and MDA-MB-436) were obtained from FuDan IBS Cell Center, China. And the human mammary epithelial cell line (MCF-10A) was obtained from Shanghai Bioleaf Biotech Co.,Ltd, China. Cell lines were cultured in appropriate media supplemented with 10% fetal bovine serum (FBS, GIBCO Life Technologies, USA) and 1% antibiotic/antimycotic solution (Sigma-Aldrich, USA) at 37 °C in a humidified atmosphere containing 5% CO_2_: Dulbecco’s modified Eagle’s medium (DMEM; GIBCO Life Technologies, USA) for SK-BR3, MCF-7 and MCF-10A; RPMI-1640 (GIBCO Life Technologies, USA) for BT-474 and MDA-MB-231; and Leibovitz’s L-15 (GIBCO Life Technologies, USA) for MDA-MB-436.

### IHC

Paraffin-embedded consecutive sections were subjected to IHC staining for the expression of eIF3m by the rabbit polyclonal anti-eIF3m antibody (bs-9033R, Beijing BIOSS, China) diluted at 1:100 in phosphate-buffered saline (PBS, GIBCO Life Technologies, USA), with a SP Rabbit & Mouse HRP Kit (CWBIO, China). PBS without primary antibodies was used as negative control. Two pathologists independently evaluated the scores of the staining intensity of eIF3m expression (x = 0, no staining of cells; 1, mild staining; 2, moderate staining; and 3, marked staining). Any disagreement was resolved by discussion. Scores of the percentage of cells were measured by ImageJ 1.52r (y = 0%–100%). The total score (immunoreactivity score, IRS = x×y × 100) ranged from 0 to 300.

### Cell transfection

To knock down eIF3m expression, pLKO.1-eIF3m-shRNA-puro was used, and three shRNAs were designed as listed in Table 1. The titer of lentivirus was 1x10^8^ TU/ml. MDA-MB-231 and MDA-MB-436 were cultured for 24 h in a 24-well plate, and then cells were transfected with shRNAs-Puro lentivirus (100 μl/ml) in a medium of 8 mg/ml polybrene (Sigma-Aldrich, USA) when a confluence of 30–50%. After 24 h, transfected cells were cultured in a new medium with 1.0 μg/ml puromycin (TargetMol, China) for about 12 days. To select the most efficient shRNA, the transfection efficiency of each shRNA was analyzed through western blotting and RT-qPCR. Cells transfected with this shRNA were named as “eIF3m-shRNA” groups, and cells transfected with empty vector were named as “Vector” groups.

**Table 1:** The design of shRNA-eIF3m. SC shRNA: the control shRNA

EIF3m	Direction	Sequence (5′ to 3′)
SC shRNA	Forward	CCGGGCGATGTGGCGAACTGACACGCTCGAGCGTGTCAGTTCGCCACATCGCTTTTTG
	Reverse	AATTCAAAAAGCGATGTGGCGAACTGACACGCTCGAGCGTGTCAGTTCGCCACATCGC
shRNA #1	Forward	CCGGCAGTGTATTGCAGCCTTATTACTCGAGTAATAAGGCTGCAATACACTGTTTTTG
	Reverse	AATTCAAAAACAGTGTATTGCAGCCTTATTACTCGAGTAATAAGGCTGCAATACACTG
shRNA #2	Forward	CCGGCTTCAGATTGGAGCTGATGATCTCGAGATCATCAGCTCCAATCTGAAGTTTTTG
	Reverse	AATTCAAAAACTTCAGATTGGAGCTGATGATCTCGAGATCATCAGCTCCAATCTGAAG
shRNA #3	Forward	CCGGGACTGGAATCTCACCACTGAACTCGAGTTCAGTGGTGAGATTCCAGTCTTTTTTG
	Reverse	AATTCAAAAAGACTGGAATCTCACCACTGAACTCGAGTTCAGTGGTGAGATTCCAGTCT

### RT-qPCR

Cells and tissues were collected to isolate total RNA through Trizol regent (Thermo Fisher Scientific, USA). A total of 2 µg RNA of each sample was reverse transcribed using the SuperScript II RNase-Reverse Transcriptase system (Thermo Fisher Scientific, USA). cDNA was subjected to quantitative PCR using primers specific for eIF3 subunits, co-expressed genes and GAPDH. PCR primers were designed as listed in Table 2 and Additional file 1: Table S1. The PCR cycling conditions were as follows: (1) 94 °C for 4 min; (2) 40 cycles of 95 °C for 1 min; (3) 60 °C for 1 min; and (4) 72 °C for 1 min. Amplifed DNA was measured by the SYBR Premix Ex Taq™ kit (Takara Bio, Japan), and qPCR was performed using an iQ5 real-time PCR detection system (Bio-Rad, USA). Then, we used 2^−ΔΔCt^ value to calculate the relative expression. The formula of ΔΔCt could refer to the previous method [14].

**Table 2:** The primer design of eIF3 subunits and GAPDH

Genes	Direction	Sequence (5′ to 3′)	Length (bp)
eIF3a	Forward	GAGCGATCATCCTGGCGTAA	332
	Reverse	GGTCTTGAGTCATCACCCCG	
eIF3b	Forward	GCACTGTGGGGACGGAC	261
	Reverse	TGGAAGCGGGTGCCTTAAAT	
eIF3c	Forward	GGGCTCAGCTGGTTGGTATT	207
	Reverse	CCGAACCGGTGGTGAAAAAC	
eIF3d	Forward	TCGGCTAGGAAAGGTTGCAG	217
	Reverse	TTTGTCTCTGCGGAGGTTCC	
eIF3e	Forward	TGCAAGACTGGATGCCAAGA	185
	Reverse	TTGCCCAGTTAGGAGCCTCT	
eIF3f	Forward	CTGCACCCAGTCATTTTGGC	297
	Reverse	TCGGCTGTAGTACTCGTGGA	
eIF3g	Forward	GAGACCCGGAAGGCTTCAAA	205
	Reverse	ACACGATCTTCTGGCCCTTG	
eIF3h	Forward	AAAACAAGCCCTGACCGGAA	187
	Reverse	TCATTTGGGAGCAGCGAAGT	
eIF3i	Forward	CGGGATGAAGCCGATCCTAC	316
	Reverse	CCCAAAGTCAAAACCGCAGG	
eIF3j	Forward	GGACGTCAAGGATAACTGGGA	324
	Reverse	TCTCTTGAAGATGGGTTCATAGCA	
eIF3k	Forward	TGCTCAAGGGTATCGACAGG	267
	Reverse	AAATCTGTCGGATTGGCCGT	
eIF3l	Forward	AAGCCATTGCTCCACAGGTT	273
	Reverse	TCTTGGCAGTCTTACAGCGG	
eIF3m	Forward	GACATCAGTGAAGAAGATCAGGC	150
	Reverse	ATCATCCTCCTTCAGACACACA	
GAPDH	Forward Reverse	GAAGGTGAAGGTCGGAGT	226
		GAAGATGGTGATGGGATTTC	

### Cell viability assay

To assess cell proliferation, 2000 stable transfected cells per well were seeded in 96-well plates for 1–5 days, and then were treated with CCK-8 (cell counting kit-8, Immunoway, USA) for 2 h at the incubator. The absorbance of each group was read at 450 nm of the microplate reader (OD value). Cell viability was calculated by the formula: Cell viability (%) = OD value of “eIF3m shRNA”/OD value of “Vector”.

### Colony formation assay

Cells were seeded in 6-well plates at a density of 200 cells. They were cultured for about 10 days, until the clones were visible (≥ 50 cells). The colonies were fixed for 15 min with methanol and then stained with crystal violet for 15 min after airing. Finally, numbers of colonies were counted.

### Cell cycle analysis

Centrifugated cells were washed 2 times with pre-cooling PBS. And then, they were fixed in 80% ethanol for about 12 h at 4 °C. Cells were washed 2 times again with pre-cooling PBS prior to incubation for 30 min at 37 °C in dark with propidium iodide and RNAse A (KeyGen Biotech, China). Fluorescence-activated cell sorting analysis was performed in the flow cytometer (Becton, Dickinson and Company, USA).

### Wound healing assay

Transfected cells were planted into 6-well plates to complete confluence in order to assess the role of eIF3m on the migration. Cells were wounded by scratching with a sterile plastic 200 µL micropipette tip and the loose cells were removed by PBS. Subsequently, Opti-MEM medium (GIBCO Life Technologies, USA) was added to the wells for incubation at 37 °C with 5% CO_2_ for 24 h. Finally, images were captured by the inverted microscope (IX73, Olympus, Shanghai, China). The relative proportion of wound = the width of the wound/the initial width.

### Transwell assays

8 mm transwell inserts (JETbiofil, China) were placed into 24-well plates, separating upper and lower chambers. The upper side of the membrane was precoated with Matrigel (Corning, USA) for gel formation. New medium was added to the lower chamber and 10^5^ cells/well in Opti-MEM were added to the upper chamber. After 72 h at 37 °C, the number of invading cells was counted through a counting chamber under the inverted microscope.

### Mitochondrial membrane potential assay

This assay could be divided into four parts:

1. Microscopic observation: Transfected cells were centrifugated and add with JC-1 working solution (Beyotime Biotechnology, China) for cultivation at 37 °C for 20 min. After washing and centrifugation by JC-1 diluent (1 ×) for 2 times, the red and green fluorescence were detected by fluorescence microscope (Olympus Corporation, China).
2. Flow cytometric analysis: Then, centrifugated cells that were treated by JC-1 were analyzed with the flow cytometer.
3. Purified mitochondria: Mitochondrial protein and cytosol were separated by Minute™ Mitochondria Isolation Kit (Invent Biotechnologies, Inc., USA). JC-1 kit (Beyotime Biotechnology, China) was used to analyze the level of red fluorescence and green fluorescence in mitochondrial proteins through the microplate reader. The ratio of red to green fluorescence was used to measure the level of depolarization of mitochondria.
4. Cyto-c releasing: Cyto-c (1:1000, ABclonal Technology, China) in mitochondria or cytosol was investigated by western blotting. In addition, COX-IV and Tubulin (1:1000, ABclonal Technology, China) were used as the reference protein of mitochondria and cytosol, respectively.

### Cell apoptosis analysis

Centrifugated cells were washed 2 times with PBS and stained with PE and 7-AAD for 15 min at room temperature in dark (KeyGen Biotech, China). After diluted in PBS, stained cells were analyzed with the flow cytometer.

### Western blot analysis

Proteins of tissues or cells were extracted by RIPA extraction buffer with PMSF (Beyotime Biotechnology, China). 20 µL per sample were loaded in pre-cast gels (Thermo Fisher Scientific, USA) for electrophoresis. And then, proteins were transferred into PVDF membranes (Beyotime Biotechnology, China) for exposure. eIF3m, CyclinD1, CyclinE, CDK2, CDK4, CDK6, Bax, Bcl-2, Bad, E-cadherin, N-cadherin, Vimentin, Snail, Twist (1:1000, Beijing BIOSS, China), P21, P27 (1:500, ImmunoWay Biotechnology, USA), Caspase3 (1:1000, Abcam, USA), Cleaved-Caspase3 (1:500, Abcam, USA), Caspase9 (1:1000, Abcam, USA) and Cleaved-Caspase9 (1:300, Abcam, USA) were primary antibodies, while GAPDH (1:1000, Cell Signaling Technology, USA) was used as the reference protein. The gray value of each protein was investigated by ImageJ 1.52r. The relative level of each protein was deduced from the ratio of the mean value of each band to that of GAPDH.

### Statistical analysis

All experiments were done in triplicates. Continuous variables were expressed as the‾x ± SD and experiments were performed in triplicate. All results were analyzed by *t* test through SPSS 20.0 software with a *p *< 0.05 considered statistically significant. The differences of eIF3 subuntis expression between breast tumors and matched normal tissues were analyzed by a paired-*t* test, while the differences of biological behaviors between “Vector” group and “eIF3m-shRNA” group were analyzed by an unpaired-*t* test. An ANOVA followed by Tukey’s multiple comparisons test was used to compare eIF3m expression in cell lines. Kaplan–Meier analysis and the log rank test were also conducted to analyze survival data of public databases. All graphs were generated by GraphPad Prism 6.0.

## Results

### Abnormal up-regulation of eIF3m had worse prognosis in BC in multiple databases

**Fig. 1:**
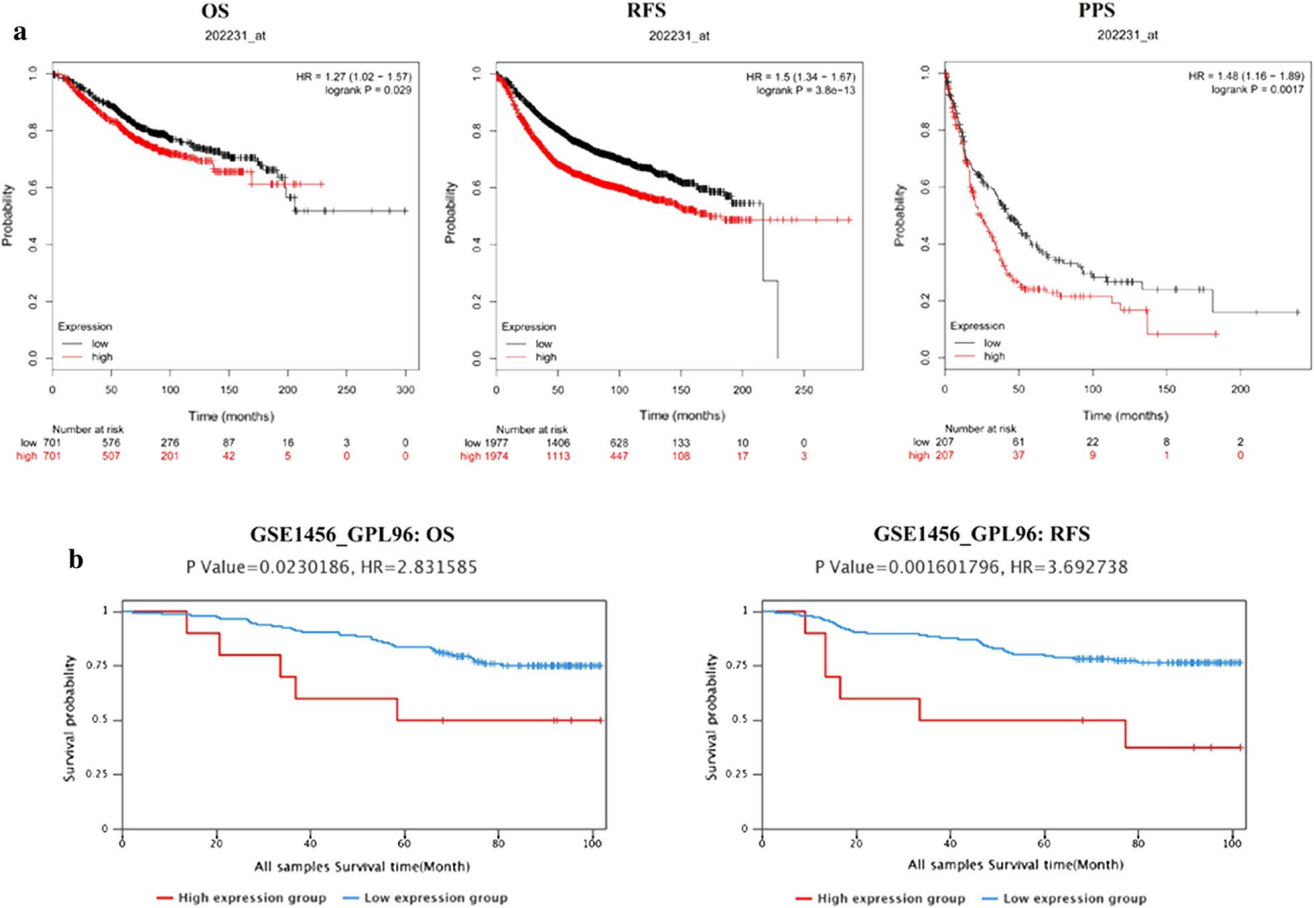
Kaplan-Meier analysis of breast cancer in public databases. **a** KM-plotter; **b** BCIP database

Oncomine analysis of tumor versus. normal samples revealed that eIF3m was significantly elevated in invasive breast carcinoma, such as invasive ductal breast carcinoma, mixed lobular and ductal breast carcinoma, invasive lobular breast carcinoma, and so on (Table 3). In addition, there were no substantial differences of eIF3m between normal breast tissues and benign breast neoplasm (*p *> 0.05, Table 3). KM-plotter and BCIP database revealed that high eIF3m was correlated with a poorer prognosis, including OS, RFS and PPS in BC (Fig. 1).

**Table 3:** Comparison of eIF3m expression between different subtypes of breast cancer and breast

Author	Subtypes (sample)	*P*-value	Fold change
Curtis	IBC vs. Breast (21 vs. 144)	4.30E − 6	1.801
	BPT vs. Breast (5 vs. 144)	0.008	1.828
	TBC vs. Breast (67 vs. 144)	7.17E − 14	1.922
	IDLBC vs. Breast (90 vs. 144)	2.87E − 12	1.844
	IDBC vs. Breast (1556 vs. 144)	7.49E − 16	1.442
	BC vs. Breast (14 vs. 144)	0.024	1.284
	MBC1 vs. Breast (46 vs. 144)	6.08E − 8	1.635
	DBCS vs. Breast (10 vs. 144)	0.005	1.412
	MBC2 vs. Breast (32 vs. 144)	3.24E − 4	1.593
	ILBC vs. Breast (148 vs. 144)	4.64E − 7	1.338
	BBN vs. Breast (3 vs. 144)	0.057	1.437
Sorlie	DBC vs. Breast (92 vs. 4)	0.037	1.169
Ma	IDBC vs. Breast (9 vs. 14)	0.016	1.102
Richardson	DBC vs. Breast (40 vs. 7)	0.002	1.182
TCGA	IDBC vs. Breast (639 vs. 111)	9.24E − 5	1.030

*IBC* invasive breast carcinoma, *BPT* breast phyllodes tumor, *TBC* tubular breast carcinoma, *IDLBC* mixed invasive ductal and lobular breast carcinoma, *IDBC* invasive ductal breast carcinoma, *BC* breast carcinoma, *MBC1* mucinous breast carcinoma, *DBCS* ductal breast carcinoma in situ; *MBC2* medullary breast carcinoma, *ILBC* invasive lobular breast carcinoma, *BBN* benign breast neoplasm, *DBC* ductal breast carcinoma

### eIF3m became a unique and valuable indicator for TNBC

**Fig. 2:**
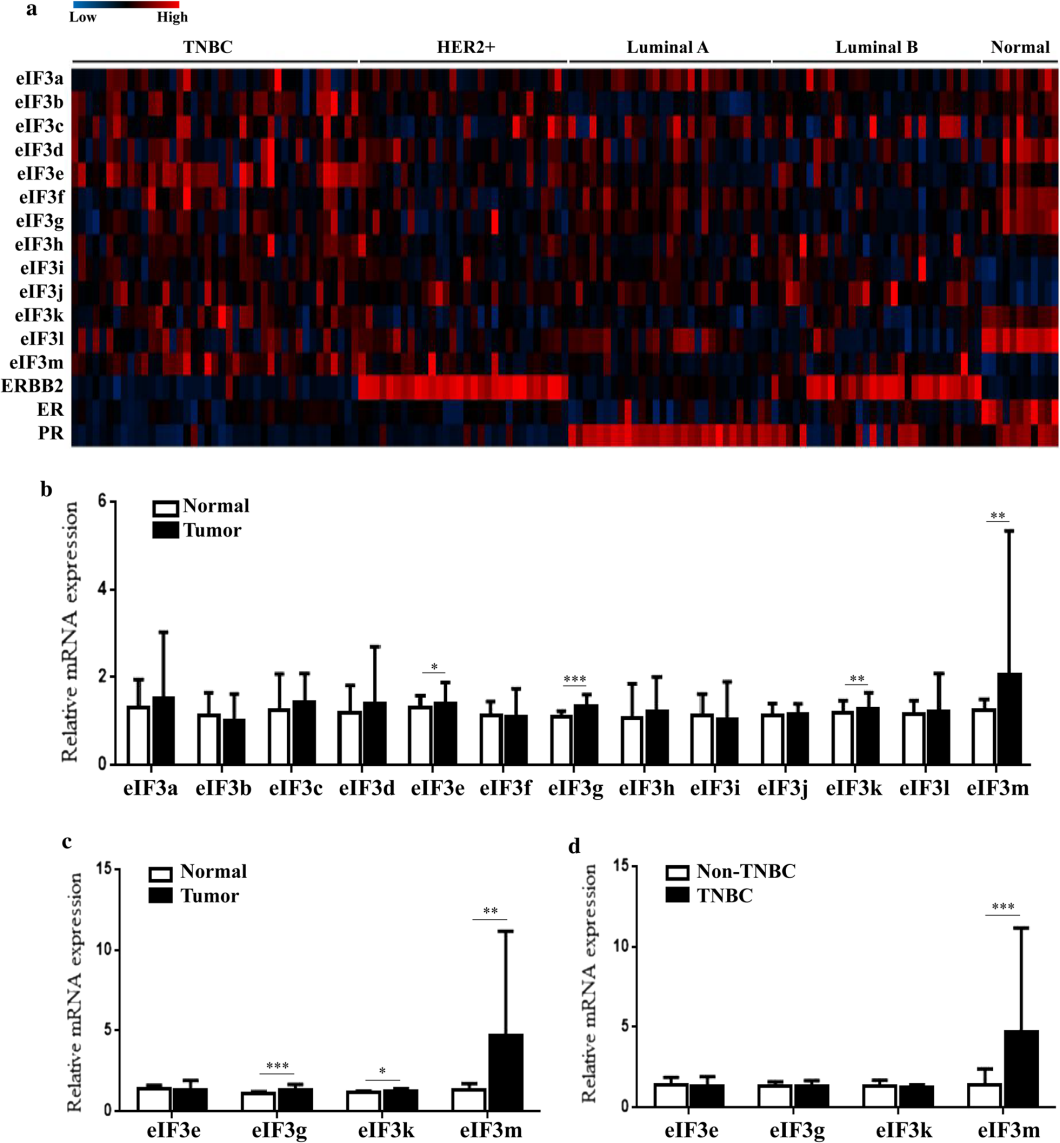
Expression of eIF3 subunits in different subtypes of breast cancer and breast normal tissues. **a** GEO database (GEO accession: GSE45827); **b** Comparison of 13 subunits expression between breast tumors and corresponding adjacent normal tissues by RT-qPCR (n = 158); **c** Comparison of subunits expression between TNBC and corresponding adjacent normal tissues by RT-qPCR (n = 32); **d** Comparison of subunits expression between TNBC (n = 36) and non-TNBC (n = 126) by RT-qPCR. TNBC: triple negative breast cancer. *p < 0.05, **p < 0.01, ***p < 0.001

To analyze differences of eIF3m expression among different types of breast cancer, one GEO database (GEO accession: GSE45827) showing all subunits expression in TNBC, non-TNBC and normal-like tissues was performed (Fig. 2a). In Table 4, expression levels of eIF3b, d, f, g, i, j, k, l and m in breast tumors were significantly higher than in normal-like tissues (*p *< 0.05). Among these subunits, there were no differences of eIF3b and k expression between TNBC and normal-like tissues, or between TNBC and non-TNBC (*p *> 0.05). Only eIF3m expression in TNBC was obviously higher than in non-TNBC tissues (*p *= 0.000253). In addition, similar results were found in 158 BC cases via RT-qPCR (Fig. 2b–d). These results reflected that eIF3 was a vital protein molecule in BC, and eIF3m was involved in the occurrence and development of TNBC.

**Table 4:** Comparison of eIF3 subunits expression among different groups of samples in GSE45827

Genes Genes *p*-value Types	Tumor vs. Normal	TNBC vs. Normal	TNBC vs. Non-TNBC
eIF3a	8.94E − 02	5.79E − 02	3.24E − 02*
eIF3b	2.83E − 02*	6.44E − 01	8.15E − 05*
eIF3c	8.54E − 01	6.16E − 01	1.34E − 01
eIF3d	1.28E − 05*	7.21E − 04*	4.55E − 01
eIF3e	7.67E − 01	4.05E − 02*	6.60E − 08*
eIF3f	5.29E − 04*	4.86E − 03*	5.77E − 01
eIF3g	4.56E − 07*	1.01E − 04*	3.03E − 01
eIF3h	3.10E − 01	8.88E − 02	1.04E − 01
eIF3i	1.12E − 07*	4.86E − 07*	4.12E − 01
eIF3j	4.28E − 17*	1.72E − 09*	2.72E − 01
eIF3k	3.46E − 05*	5.20E − 02	6.85E − 06*
eIF3l	8.20E − 13*	5.59E − 09*	9.46E − 01
eIF3m	1.10E − 06*	2.01E − 07*	2.53E − 04*

*TNBC* triple negative breast cancer, *Non-TNBC* other subtypes of breast cancer except triple negative breast cancer

*p-value was significant

**Fig. 3:**
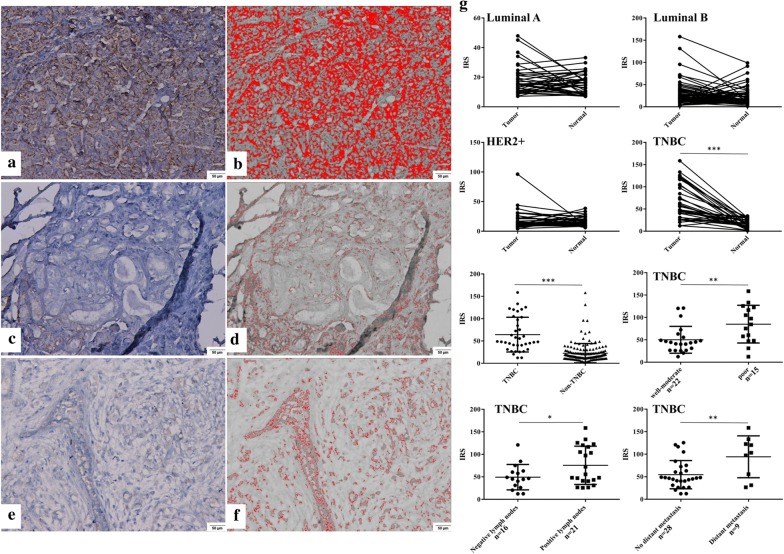
eIF3m expression in breast tumors and normal tissues via immunohistochemistry. a, c, e. eIF3m expression in poor (**a**) and well-moderate (**c**) differentiation of TNBC and normal tissues (**e**) were investigated at × 200 magnification; **b, d, f** ImageJ analysis of eIF3m expression (× 200); **g** Analysis of eIF3m expression in different subtypes of breast tumor and normal tissues, as well as in differentiation, lymph node invasion and distant metastasis. *p < 0.05, **p < 0.01, ***p < 0.001

Expression of eIF3m was detected in the cytoplasm through IHC (Fig. 3). There were no differences of eIF3m expression between tumors and corresponding adjacent normal breast tissues in non-TNBC. But, its expression levels in TNBC were significantly higher compared with adjacent normal tissues (*p *< 0.001). In addition, expression of eIF3m in TNBC was also higher than non-TNBC (*p *< 0.001).

Poor differentiation group of TNBC had a higher level of eIF3m in comparison of well-moderate differentiation group (*p *< 0.01, Fig. 3g). Cases with lymph node metastasis positive or distant metastasis positive group also had a higher level of eIF3m compared with metastasis negative group (*p *< 0.05, *p *< 0.01, Fig. 3g).

### eIF3m expression in breast cell and breast cancer cell lines

**Fig. 4:**
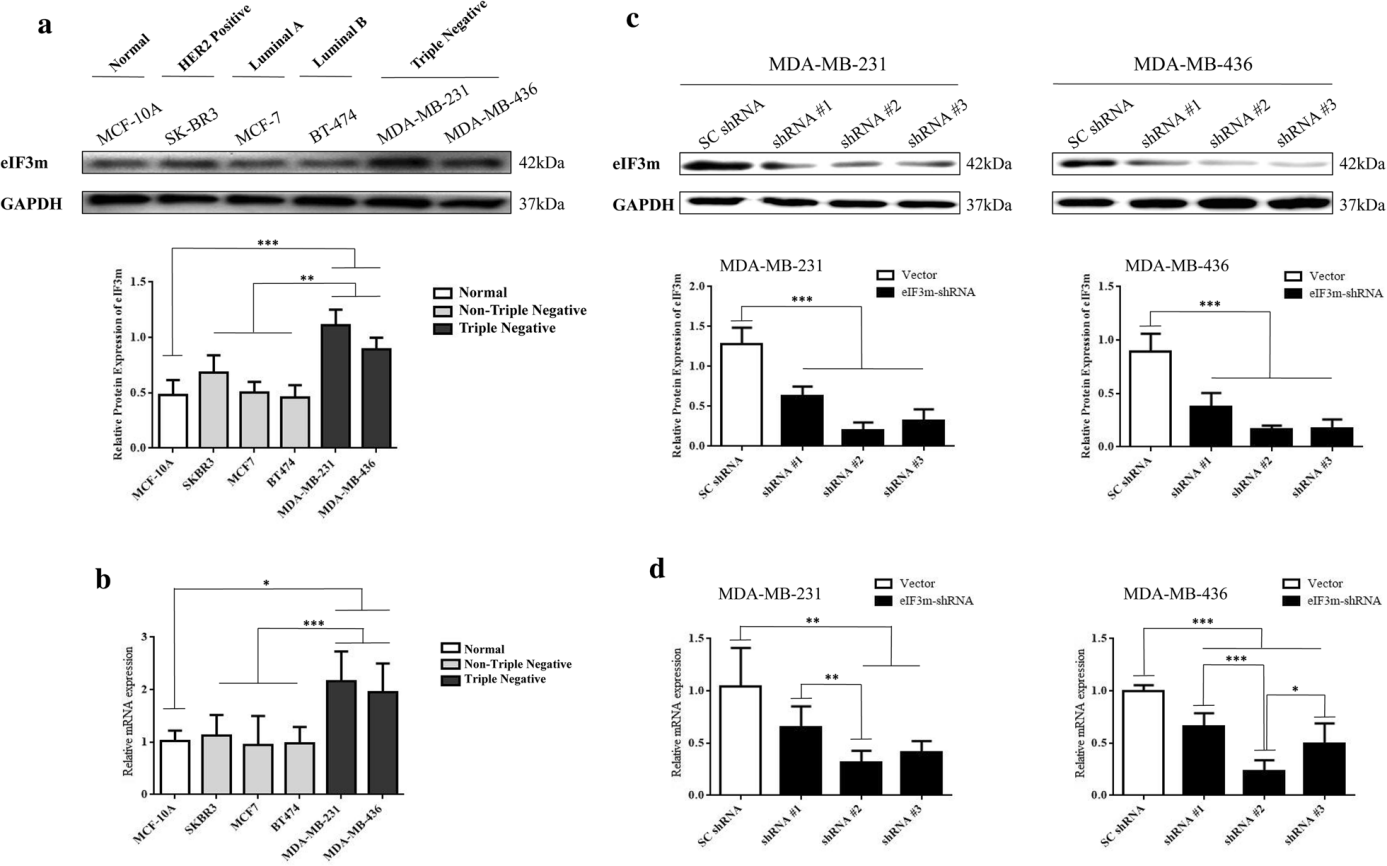
eIF3m expression in cell lines. **a, b** Expression of eIF3m protein (**a**) and mRNA (**b**) in breast cancer cells and MCF-10A was investigated via western blotting and RT-qPCR, respectively; **c, d** Regulation of eIF3m protein (**c**) and mRNA (**d**) in transfected cells (MDA-MB-231 and MDA-MB-436) was analyzed through western blotting and RT-qPCR, respectively. *p < 0.05, **p < 0.01, ***p < 0.001

Among five BC cell lines and MCF-10A, MDA-MB-231 and MDA-MB-436, two TNBC cell lines, showed highest expression of eIF3m (Fig. 4a, b). Therefore, these two cell lines were chosen to be transfected with lentivirus. The transfection efficiency was accessed by western blotting (Fig. 4c) and RT-qPCR (Fig. 4d). Among shRNAs, shRNA2# had the lowest eIF3m expression. Thus, we used shRNA2# for the next assays.

### Knocking down eIF3m inhibited cell proliferation in TNBC cells

**Fig. 5:**
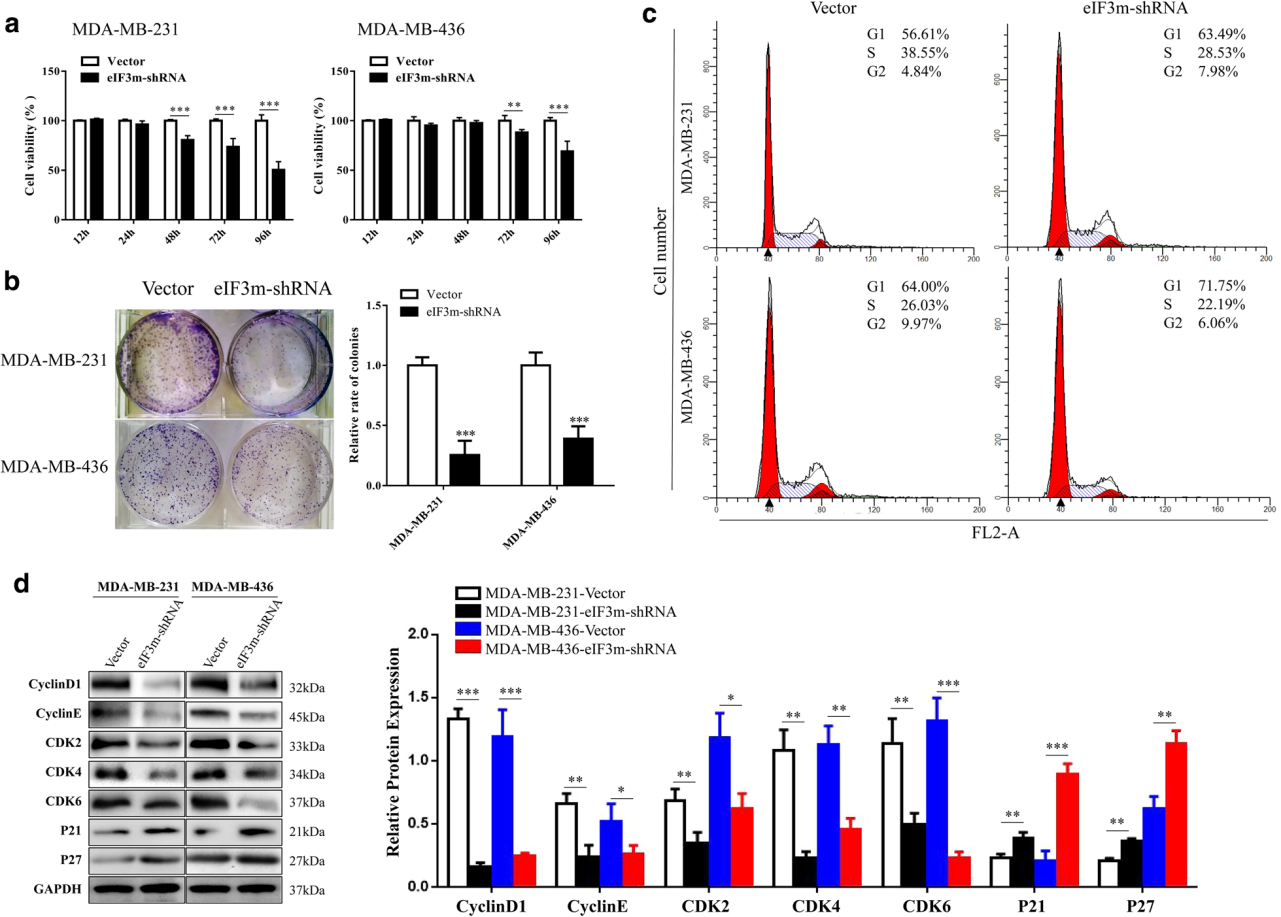
Influence of eIF3m knockdown on cell proliferation in TNBC. **a** The viability of transfected cells was determined by CCK-8 assay at 450 nm OD value of the microplate reader; **b** The effects of eIF3m on cell growth were confirmed by colony formation assay; **c** Cell cycle distributions were detected by flow cytometric analysis in transfected cells; **d** Cell cycle-related proteins of transfected cells were investigated via western blotting. *p < 0.05, **p < 0.01, ***p < 0.001

As shown in Fig. 5a, reduced expression of eIF3m obviously suppressed cell viability (*p *< 0.05). The number of colonies formed on 6-well plates in “eIF3m-shRNA” groups was significantly fewer than in “Vector” groups (*p *< 0.01, Fig. 5b). In addition, cells transfected with eIF3m-shRNA obtained a higher proportion at G1 phase (Fig. 5c). At the same time, expression of CyclinD1, CyclinE, CDK2, CDK4 and CDK6 decreased obviously in “eIF3m-shRNA” groups in comparison of “Vector” groups; but P21 and P27 in “eIF3m-shRNA” groups were up-regulated (Fig. 5d). These results confirmed that knockdown of eIF3m suppressed proliferation of TNBC cells.

### Knocking down eIF3m suppressed migration and invasion in TNBC cells

**Fig. 6:**
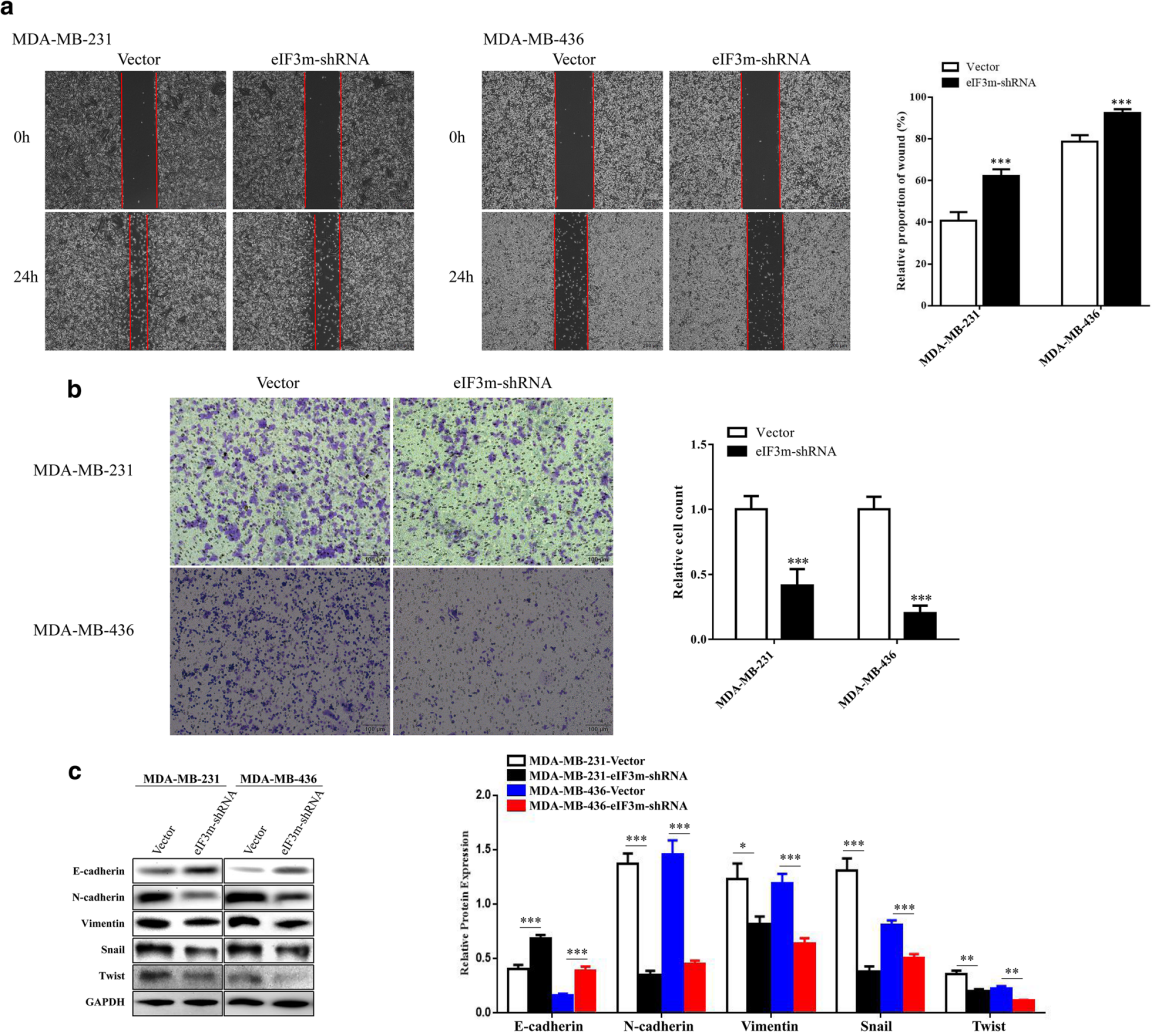
Influence of eIF3m knockdown on migration and invasion in TNBC. **a** Wound healing assay measured relative proportion rates at 24 h for comparing migration abilities of different cells (× 40); **b** Transwell assay detected the number of cells passing through chambers in each group by image analysis (× 100); **c** Epithelial–mesenchymal transition related proteins of transfected cells were investigated via western blotting. *p < 0.05, **p < 0.01, ***p < 0.001

By knocking down eIF3m, the width of scratch area after 24 h was obviously longer compared with “Vector”, (Fig. 6a). Also, the transwell assay showed fewer cells going through chambers when decreasing expression of eIF3m in TNBC (Fig. 6b). Knockdown of eIF3m debased the levels of N-cadherin, Vimentin, Snail and Twist, while it elevated E-cadherin expression (Fig. 6c). Thus, low expression of eIF3m made a sharp decline in the abilities of migration and invasion.

### Knocking down eIF3m promoted apoptosis in TNBC cells

**Fig. 7:**
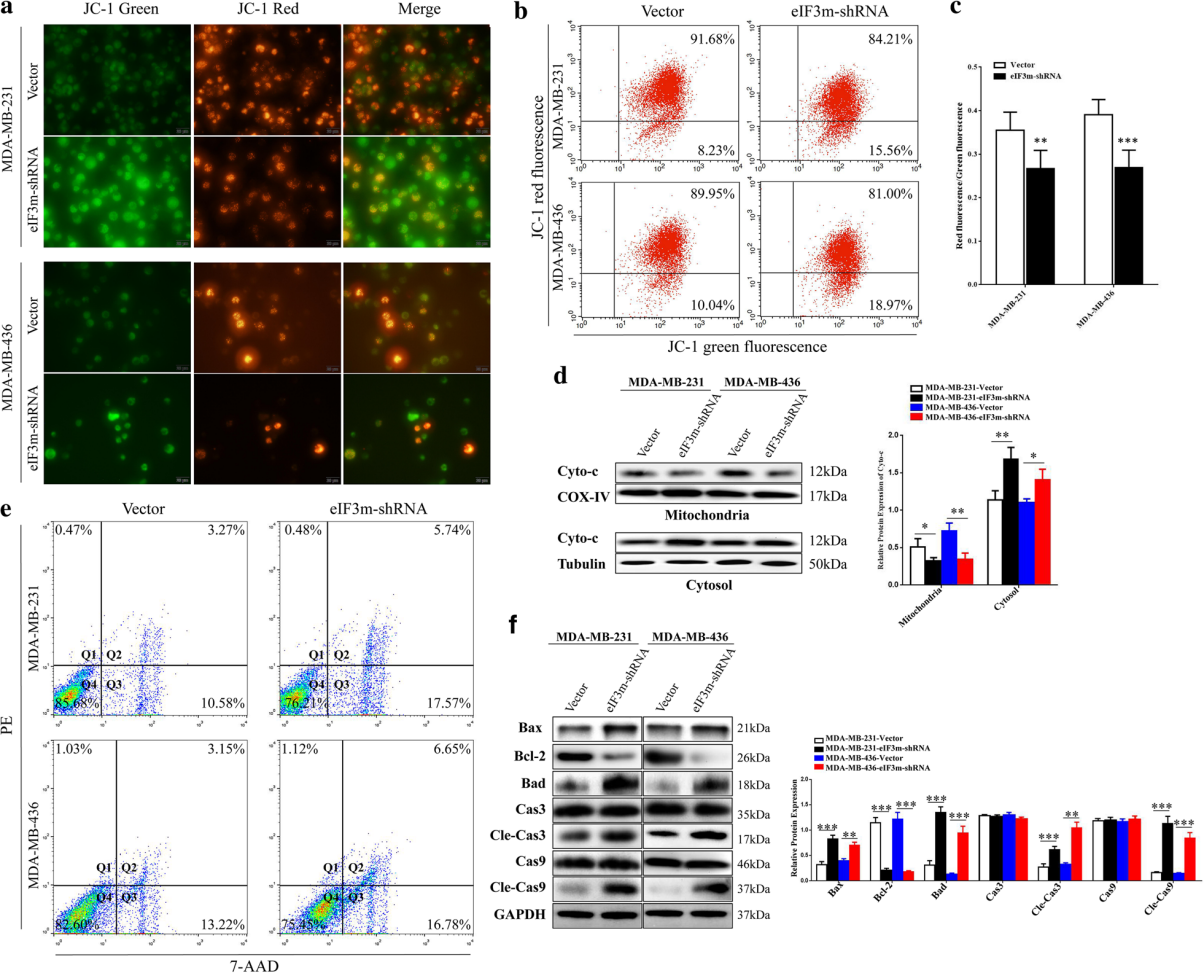
Influence of eIF3m knockdown on mitochondrial membrane potential and cell apoptosis in TNBC. **a** JC-1 green and JC-1 red of cells were observed in the fluorescence microscope (× 400); **b** JC-1 green and JC-1 red distributions were detected by flow cytometric analysis; **c** ratios of red/green fluorescence in mitochondria were measured through the microplate reader; **d** Blots and relative protein expression of Cyto-c expression in mitochondria and cytosol of stably transfected cells; **e** Cell apoptotic rates were detected by flow cytometry; **f** Apoptosis-related proteins of transfected cells were investigated via western blotting, Cle-Cas3: Cleaved-Caspase-3; Cas3: Caspase-3; Cle-Cas9: Cleaved-Caspase-9; Cas9: Caspase-9. *p < 0.05, **p < 0.01, ***p < 0.001

The brightness of JC-1 red in MDA-MB-231 and MDA-MB-436 was reduced by using eIF3m-shRNA, while JC-1 green’s brightness was increased obviously (Fig. 7a).The ratios of red/green fluorescence were also significantly decreased (Fig. 7b, c). Through extraction of mitochondrial proteins and cytosol, knockdown of eIF3m down-regulated Cyto-C obviously in mitochondria, but elevated its expression in cytosol (Fig. 7d). In addition, rates of cell apoptosis in “eIF3m-shRNA” groups were increased along with the regulation of Bcl-2 family and the activation of Caspase-3 and Caspase-9 (Fig. 7e, f). These results revealed that knockdown of eIF3m elevated apoptosis rates through releasing Cyto-C from mitochondria into cytosol and activating Caspases.

### Functions of eIF3m on the development of breast and TNBC analyzed by databases

**Fig. 8:**
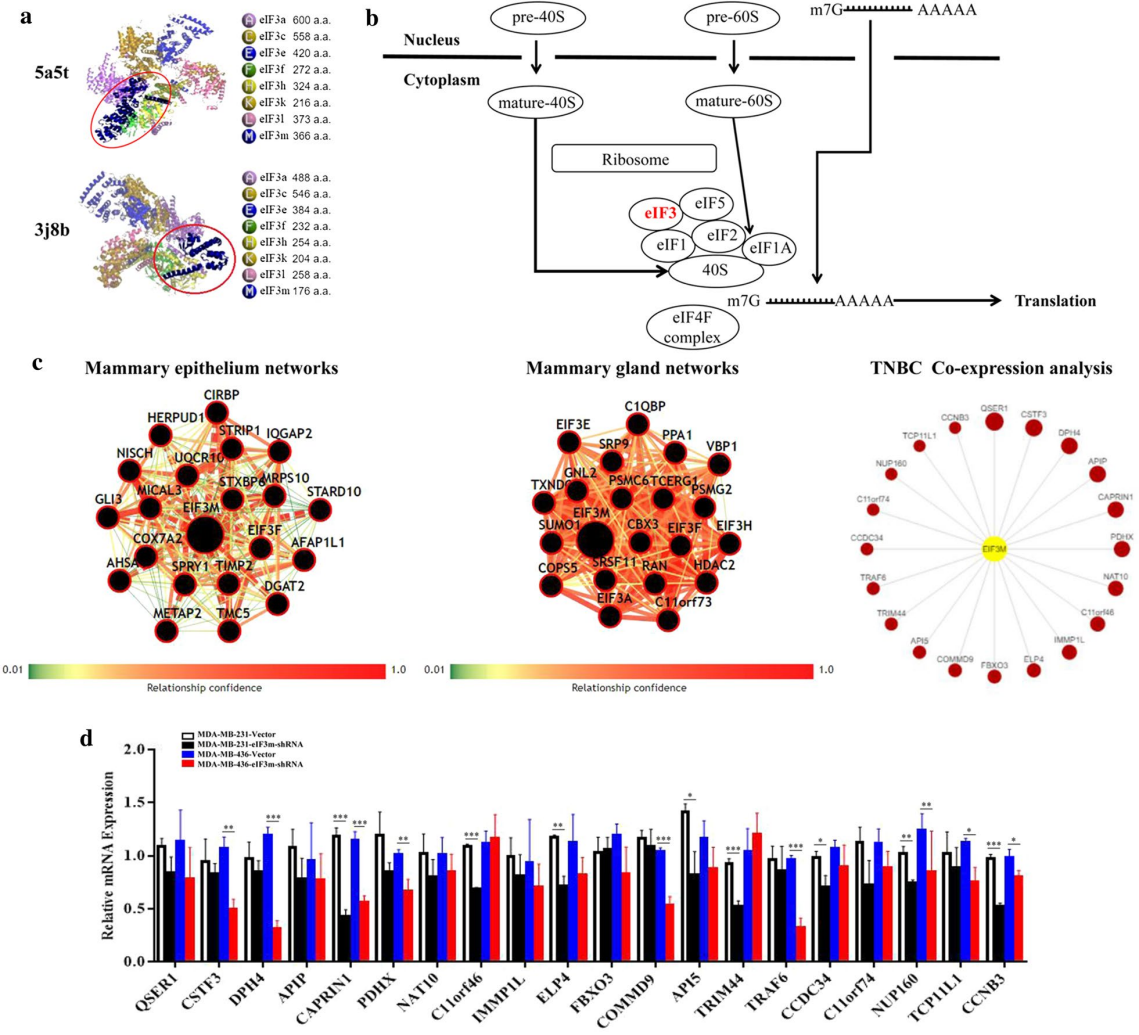
Functions of eIF3m on the development of breast and TNBC in public databases. **a** PDBsum entry displayed the structure of eIF3; **b** Schematic model of the role of eIF3 on RNA transport analyzed by KEGG pathways; **c** BCIP database showed co-expressed genes of eIF3m in the development of breast and TNBC; **d** RT-qPCR investigated co-expressed genes in different groups. *p < 0.05, **p < 0.01, ***p < 0.001

**Fig. 9:**
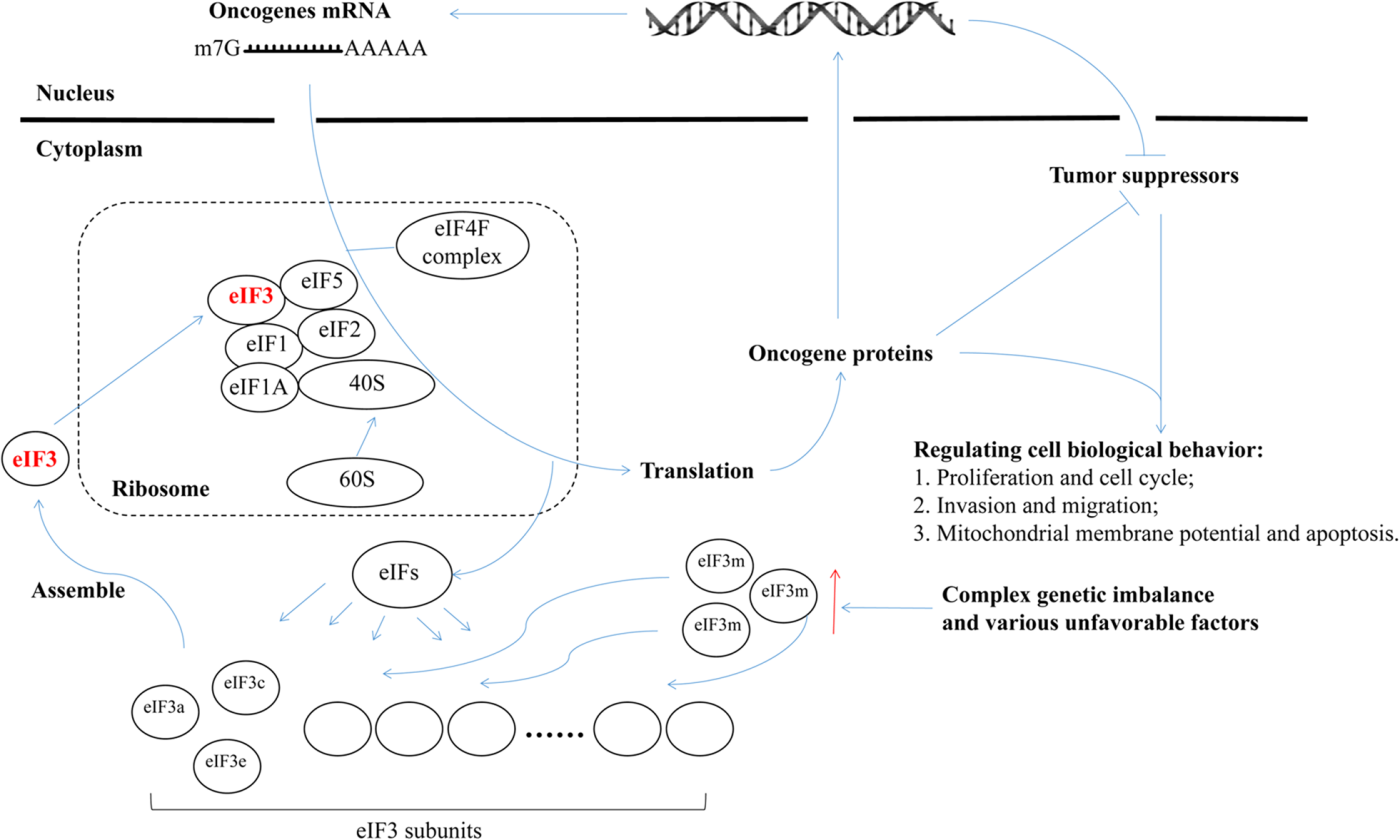
Schematic model of the carcinogenic mechanism of eIF3m in TNBC

PDBsum entry showed the structure of eIF3, and eIF3m was a key subunit maintaining the integrity of the eIF3 complex by stabilizing other subunits (Fig. 8a) [15, 16]. KEGG pathways displayed the vital role of eIF3 on RNA transport in which eIF3 and other eukaryotic translation initiation factors must be combined with the ribosome and mRNAs in order to complete the translation (Fig. 8b). In addition, eIF3m was involved in the development of mammary epithelium and gland (Fig. 8c). During the development of TNBC, eIF3m became an essential factor of the activation of various genes (Fig. 8c). Through RT-qPCR, 14 of top 20 co-expressed genes, most of which were oncogenes, including CSTF3, DPH4, CAPRIN1, PDHX, C11orf46, ELP4, COMMD9, API5, TRIM44, TRAF6, CCDC34, NUP160, TCP11L1 and CCNB3, had lower expression in “eIF3m-shRNA” groups than in “Vector” groups (Fig. 8d and Table 5). Therefore, the carcinogenic mechanism of eIF3m was displayed in Fig. 9.

**Table 5:** Functions/expression of genes co-expressed with eIF3m in triple negative breast cancer from BCIP database

Genes	Correlation coefficient	Qvalue	Functions/expression in cancers	RefSeq ID
QSER1	0.714601888	9.34E − 15	Over-expressed in ovarian cancer [17]	NM_024774
CSTF3	0.672936196	5.44E − 12	Over-expressed in colorectal cancer [18]	NM_001326
DPH4	0.653121381	6.09E − 11	Protein biosynthesis related with eEF2 [19]	NM_181706
APIP	0.652049629	6.09E − 11	an ERBB3-binding partner promoting tumorigenesis [20]	NM_015957
CAPRIN1	0.638113822	3.09E − 10	Carcinogenesis in breast cancer [21]	NM_005898
PDHX	0.635217128	3.80E − 10	Regulating glucose metabolism [22]	NM_003477
NAT10	0.603723528	1.29E − 08	Epithelial-mesenchymal transition [23]	NM_024662
C11orf46	0.594934276	2.94E − 08	Growth of the corpus callosum [24]	NM_152316
IMMP1L	0.591451963	3.81E − 08	Strongly implicated in obesity [25]	NM_144981
ELP4	0.556089434	1.06E − 06	Promoting the migration and invasion of hepatocellular carcinoma [26]	NM_019040
FBXO3	0.545140598	2.56E − 06	Regulating p53 transcriptional activity [27]	NM_012175
COMMD9	0.538715159	4.09E − 06	Promoting non-small cell lung cancer cancer progression via the activation of TFDP1/E2F1 transcriptional [28]	NM_014186
API5	0.523379598	1.34E − 05	Anti-apoptotic factor of breast cancer [29]	NM_006595
TRIM44	0.517213906	2.03E − 05	Promoting proliferation and invasion of thyroid cancer via Wnt/β-catenin [30]	NM_017583
TRAF6	0.513110012	2.62E − 05	Ubiquitinating DNA methyltransferase proteins [31]	NM_145803
CCDC34	0.501851465	5.76E − 05	Carcinogenesis in colorectal cancer [32]	NM_030771
C11orf74	0.48986957	0.000129328	Interacting with the IFT-A complex [33]	NM_138787
NUP160	0.484247975	0.000181588	Oncogene in angiosarcoma [34]	NM_015231
TCP11L1	0.477552672	0.000272389	Involved in heterozygous deletion and related with eIF3m [35]	NM_018393
CCNB3	0.476854234	0.000272389	Fused with BCOR and involved in cancer [36]	NM_033031

*Qvalue* the percentage of the mistaken ones in all individuals with significant differences

## Discussion

It is well known that breast tumor occurs in the process of continuous and cumulative improper regulation of gene expression. This abnormal regulation is caused by various exterior and internal carcinogenic factors. In other words, molecular mechanisms of the BC tumorigenesis are not thoroughly understood.

EIFs are involved in translation and protein synthesis in order to meet the demand of successful embryonic development and maintenance of normal tissue homeostasis [37]. Among them, several proteins or subunits of EIFs are essential for tumorigenesis [5]. Recently, oncologists have focused on the biggest eukaryotic translation initiation factor, eIF3, which is constituted by 13 subunits. This molecule participates in the 43S pre-initiation complex formation and facilitates recruiting GTP-eIF2-tRNA-methionine ternary complex [38]. The complex stimulates mRNA binding with 43S pre-initiation complex, promotes the process of translation and influences cell biological behaviors and functions [39]. Not only each subunit plays an important role in the synthesis and metabolic activities, but also several ones are implicated in carcinogenesis. As the largest subunit, eIF3a has vital biological functions in various carcinomas, including BC [40]. Other core subunits, eIF3c, g and i are also associated with BC tumorigenesis, especially in drug resistance [11, 41, 42]. In addition, several “non-core” subunits (eIF3b, d, e and f) correlated strongly with the tumorigenesis of breast neoplasm [43–46].

Based on public databases, a unique advantage of eIF3m could be found in TNBC. Among 13 subunits, only eIF3m met the three points: 1. a higher level in breast tumor than in normal tissue; 2. a higher level in TNBC than normal-like tissues; and 3. a higher level in TNBC than in non-TNBC. These results revealed that eIF3m might become a critical molecule in the development and progression of BC, especially in TNBC. One previous study demonstrated that eIF3m was required for cell-cycle and cell proliferation [14]. Silencing eIF3m almost completely turned a caspase substrate, PARP1, into activated form, which led to promoting cell death of HCT-116 [13]. In this research, we verified functions of eIF3m on cell-cycle and cell apoptosis of TNBC. Through regulation of CyclinD1 and other cell-cycle proteins, knockdown of eIF3m arrested cell-cycle at G1 phase and inhibited cell proliferation. Decreased eIF3m also caused a sharp decline of mitochondrial membrane potential, followed by release of Cyto-C from mitochondria to cytosol. As a result, instable mitochondrial membrane activated Caspases and then induced cell apoptosis. In addition, knocking down eIF3m suppressed migration and invasion of TNBC through regulation of epithelial–mesenchymal transition (EMT) related proteins. EMT is the process of epithelial cells transformed into mesenchymal cells. It elevates motility, migration and invasion of cancer cells, leading to metastasis of tumors, especially in TNBC [47]. Thus, we argue that over-expressed eIF3m promotes proliferation and activate EMT in TNBC.

Results of IHC uncovered that high eIF3m was involved in poor differentiation, lymph node metastasis and distant metastasis of TNBC. In addition, high expression of eIF3m had a poorer prognosis of BC patients both in two public databases, KM-plotter and BCIP database. Due to no enough survival data of this cohort, further follow-ups and researches will be performed to analyze the prognostic role of eIF3m in TNBC patients. Thus, we demonstrate that eIF3m is a novel and reliable bio-marker of prognosis and clinicopathology in BC, especially in TNBC.

eIF3m maintained the integrity of the eIF3 complex in order to complete the process of translation which was essential for the development of normal breast tissues and tumors. Compared with other subtypes and normal tissues, TNBC had the highest expression level of eIF3m. This might be caused by complex genetic imbalance involving activation of oncogenes and inhibition of tumor suppressors. This imbalance was influenced by various unfavorable factors from daily life, society and environment [48]. When eIF3m was up-regulated, more oncogenes were expressed and vital signaling pathways related to tumor growth and invasion were activated. As a result, eIF3m expression was elevated again, and then improve oncogenes and pathways one more time. This complex cycle accelerated the carcinogenesis of TNBC. As for specific reasons or factors that led to eIF3m up-regulation, we still need to perform further investigations and explain potential molecular mechanisms in the near future. Due to no animal experiment temporally as a result of the limitation of our funding, the next step will include in vivo assays to identify its molecular mechanisms and roles in regulating expression of oncogenes. In addition, our research discovered a discrepancy that mRNA expression of different oncogenes was not showing the similar kind of response in these two TNBC cells. This difference might be as a result of the specificities of different cell lines or experimental errors. Therefore, further researches should choose the oncogenes with the similar response to investigate their interaction with eIF3m.

## Conclusion

eIF3m was an indispensable subunit of eIF3 and played a vital role in the process of TNBC tumorigenesis. High expression of eIF3m predicted poor prognosis in BC patients. Further researches should be conducted to reveal its possible molecular mechanisms and explore eIF3m-related oncogenes, in order to provide clues for confirming potential therapeutic targets in TNBC.

## Supplementary information

**Additional file 1: Table S1**. The primer design of co-expressed genes.

## Data Availability

All data generated or analyzed during this study are included in this published article.

## Abbreviations

TNBC: Triple negative breast cancer
BC: Breast cancer
eIF3m: Eukaryotic translation initiation factor 3m
ER: Estrogen receptor
PR: progesterone receptor
HER2: c-erbB-2
EIFs: Eukaryotic translation initiation factors
eIF3: Eukaryotic translation initiation factor 3
CDC25A: Cell division cycle 25A
OS: Overall survival
RFS: Relapse-free survival
PPS: Post progression survival
IHC: Immunohistochemistry
IRS: Immunoreactivity score
RT-qPCR: Reverse transcription-quantitative PCR
EMT: Epithelial–mesenchymal transition
