# Supplementary material for: Roles of eIF3m in the tumorigenesis of triple negative breast cancer
Source: Cancer Cell Int. 2020 Apr 29;20:141. doi: 10.1186/s12935-020-01220-z (PMC7191806; doi:10.1186/s12935-020-01220-z)
Supplement: Supplementary file 1 — Additional file 1: Table S1. The primer design of co-expressed genes. [file 12935_2020_1220_MOESM1_ESM.docx]

Table S1. The primer design of co-expressed genes.

| Genes | Direction | Sequence（5’ to 3’） | Length (bp) |
| --- | --- | --- | --- |
| QSER1 | Forward  Reverse | TGCACAAGAGTTTGCTGTCG  TGGTGCTGCTAACTGAGACG | 269 |
| CSTF3 | Forward  Reverse | CAGTGCGCACGTTGTAATCA  ACCCTTGGTTTCTCGGACAT | 368 |
| DPH4 | Forward  Reverse | AGGTTCCCCCTCCCACAATA  TTACGTGCCAGCCCTAATGG | 288 |
| APIP | Forward  Reverse | ACTGCAAATGGTCACCCTGAA  ACAAGAGAATGCTCATTCCACAA | 259 |
| CAPRIN1 | Forward  Reverse | CCCAGACCAGTCCCATCAAG  TGCTGAGTGTTCATTTGCGG | 393 |
| PDHX | Forward  Reverse | CGAAGCGTAGGGCTGGTGAA  TGGTGGTGGAGGACCTACG | 387 |
| NAT10 | Forward  Reverse | GGAGCCAGGCTTACTACGTG  TCTTTGCCGCTCAGCTACTC | 223 |
| C11orf46 | Forward  Reverse | ACCTGAGTCAGATGGAAGAACTG  TCATACAGCCACTTGCGGTC | 318 |
| IMMP1L | Forward  Reverse | GCCCGAGGAATTTCAGACCT  GGGCTGGCACGTAAAAATCC | 572 |
| ELP4 | Forward  Reverse | TATTGCCCGTGTCACAACCT  AAATGCAGTCGCTCAATGGTG | 216 |
| FBXO3 | Forward  Reverse | GAAAGAGGGTGCTCGAGAGG  GTGGGGTGGATGTACAGAGC | 556 |
| COMMD9 | Forward  Reverse | TTCCAGCTTGTCTGTGACCC  ATCATTTACTGGCCACGGCA | 458 |
| API5 | Forward  Reverse | GCAAGTGGGCCAGCATAAAG  TTTAAAGGGACATCCCCGGC | 1602 |
| TRIM44 | Forward  Reverse | GCACAGTCACATTCCCTCCT  TTACACCTTGCAGGGTCCTT | 265 |
| TRAF6 | Forward  Reverse | ACCCAGCTTTCTTTGTGTGC  TCAAGCAGATGGGGCATTCA | 319 |
| CCDC34 | Forward  Reverse | AAACCTCGTCCAGCTGCAAA  TCTGCACAGAGTTCCAAGGC | 255 |
| C11orf74 | Forward  Reverse | AGAGGGGACTCCTGGAATGT  CTGGAAGCAGCAGCAAATCC | 444 |
| NUP160 | Forward  Reverse | AGCTATCAGGGGTGACCAGT  TTCCAGCAGTAAGCCGAAGG | 200 |
| TCP11L1 | Forward  Reverse | TTCCCTGGACTTTGTCACCC  TTTCTCAGCAAAGTCGGCCT | 334 |
| CCNB3 | Forward  Reverse | AGGAGATAACCCCACGGGAA  AAACTGTTCCTCTCTCTCTTTCA | 110 |
